# Supporting meaningful public involvement and engagement in health research: a call for feedback on unsuccessful grant proposals

**DOI:** 10.1186/s40900-025-00813-0

**Published:** 2025-12-22

**Authors:** Matthias Eberl, Sienna-Mae Yates, Sarah Peddle, Jim Fitzgibbon, Sarah Hatch

**Affiliations:** 1 Division of Infection and Immunity, School of Medicine, Cardiff University, Cardiff, UK; 2 Systems Immunity Research Institute, Cardiff University, Cardiff, UK; 3 Senior Public Contributors, School of Medicine, Cardiff University, Cardiff, UK; 4 Public Contributor, Cardiff, UK; 5 Public Involvement and Engagement Team, School of Medicine, Cardiff University, Cardiff, UK

## Abstract

Funders increasingly emphasise patient and public involvement and engagement (PPIE) as an integral component of health and biomedical research. This shift reflects broader commitments to inclusivity, transparency and accountability, recognising that lived experiences enhance the relevance, feasibility and impact of research. PPIE can improve study design, recruitment and dissemination, supporting a transition from expert-led to co-created knowledge generation. However, funders sometimes provide little or no feedback on unsuccessful grant applications, typically citing capacity constraints. While some organisations offer constructive critique, others—especially smaller funders—do not, undermining the very participatory principles they aim to promote. Lack of feedback hampers researchers’ professional development and disproportionately affects early-career academics, underrepresented groups and those without strong institutional support. It also risks discouraging public contributors who invest time and emotional effort in co-developing proposals, eroding trust and diminishing willingness to engage in the future. This disconnect between expectation and communication reinforces systemic inequities and risks reducing PPIE to a symbolic gesture. Strengthening response mechanisms is both a practical necessity and a moral and ethical obligation. Constructive critique is central to scientific progress; without it, the promise of PPIE to foster a reciprocal and transformative partnership risks being undermined, at a time when public trust in science is already fragile. We believe that current funding systems need to acknowledge their responsibility to deliver meaningful feedback, even within the realities of constrained resources.

## Abstract (plain language summary)

Scientists are now expected to work closely with patients and the public when planning and carrying out studies. This is especially the case in health research. Involving people with lived experience helps make research more relevant and useful. It can also improve research questions, study design and how results are shared. However, when funding applications are rejected, some funders give little or no feedback. This makes it hard for researchers—especially those at the start of their careers or working in underrepresented areas—to learn and improve. It can also discourage members of the public who gave their time to help shape a proposal but never hear why it failed. Without proper feedback, trust in the funding process and in research itself can suffer. It risks making public involvement feel like a box-ticking exercise instead of genuine partnership. We believe that funders could help by providing even brief, clear feedback.

## Background

Unfortunately, on this occasion, your application was unsuccessful. Please note that, because of the large number of applications we received, we will be unable to provide feedback on individual submissions.

(From a rejection letter, July 2025)

In the UK, there has been a significant shift towards fostering collaborations between researchers and the communities they aim to serve, particularly in health and disease-related fields. Funders now routinely expect researchers to engage and involve members of the public, from initial conception and design through to project delivery and dissemination of the results. This trend reflects a broader commitment to democratising research, improving transparency and accountability, and ensuring that studies address public priorities and deliver real-world value [1, 2]. 

The emphasis on patient and public involvement and engagement (PPIE) in biomedical research aligns with values of inclusivity and shared responsibility, acknowledges the significance of lived experiences and recognises that those impacted by research should have a voice in shaping its direction. In fact, patient insights often help refine research questions, improve study protocols and strengthen recruitment and retention strategies, thus enhancing the relevance, feasibility and impact of the research.

Set against the growing expectations for closer public involvement in research is a critical systemic gap: funders often do not provide feedback on unsuccessful grant applications, or only insufficiently so [3]. This practice is typically justified on the grounds of capacity. While some major funders are exemplary in providing detailed and formative feedback to applicants, reviewing submissions and providing tailored responses requires time and resources that other organisations, especially small charities, simply lack. However, failure to do so weakens the very spirit and effectiveness of the participatory research they aspire to champion [4].

## The importance of constructive feedback for researchers and public contributors

For researchers—especially early-career academics or those new to PPIE—feedback is crucial for personal and professional progress. Without constructive critique, it is difficult to identify where a proposal may have fallen short, making it harder to revise and improve the project for a possible resubmission to the same funder, or elsewhere.

Moreover, the disregard for feedback undermines efforts to build meaningful relationships with stakeholders. Developing a grant proposal with a strong PPIE element involves substantial time, coordination and often emotional investment, from both researchers and members of the public [5]. When an application is rejected with little or no explanation, it can be discouraging for everyone involved but it carries particular symbolic weight for public contributors, who are often vulnerable as a consequence of their lived experience. Most of all, such a ‘silent’ rejection suggests that their contributions are seen as expendable.

A disconnect between funders’ expectations and their communication with applicants can directly harm innovation and equity. Above all, it disadvantages researchers from institutions or disciplines with less established support structures and experience, thus reinforcing systemic inequalities. Underrepresented groups in research often face barriers to engaging with academic structures [5]. This is even more so the case with researchers from low and middle income countries [6]. When researchers working on issues relevant to those communities receive little or no guidance on rejected proposals, it may hamper attempts to diversify involvement.

Finally, the feedback gap erodes trust in the funding process and in science itself. Funders are increasingly asking researchers to involve patients and the public not just as beneficiaries, but as co-creators of knowledge. This represents a fundamental cultural shift from a model of expert-led investigations to one of shared decision making. Members of the public contributing their time, expertise and/or lived experience become invested in a project [5]. To request such a contribution without a commitment to follow-up—whether a project proposal is successful or not—is ethically problematic. When the substantial efforts that go into building relationships with the public are not appreciated, it reduces involvement to a symbolic gesture, rather than a genuine partnership. Ultimately, this fosters cynicism and discourages future engagement. This development is particularly concerning in a funding climate marked by surging application volumes and plummeting success rates [7], and the explicit notion by UK Research and Innovation (UKRI) that ‘different levels of feedback may be provided on unsuccessful applications’, contingent on available resources and scheme design [8]. 

## Conclusions

The increasing emphasis on PPIE in research reflects a welcome evolution in scientific values and practices, toward more inclusive, impactful and socially responsive research [1, 2]. Many funders have in fact signed up to The Shared Commitment to Public Involvement [9] and/or strongly encourage researchers to recognise and embed the UK Standards for Public Involvement [10] in their research. One of the six standards in fact focuses on Communications, highlighting the need to have processes in place ‘to offer, gather, act on and share feedback with the public’. By establishing support structures for constructive feedback and learning, funders can help cultivate a more equitable, transparent and participatory research ecosystem—one in which all voices are heard, valued and empowered.

It is appreciated that many funders come from an ethos of wanting to spend as little as possible on running costs and as much as possible on research. At the same time, reviewers are typically unpaid and often stretched beyond reasonable capacity. In this context, withholding individualised information becomes a pragmatic, if regrettable, response to systemic overload. However, we believe that current funding systems need to acknowledge their responsibility in fostering equitable supportive research environments, even within the realities of constrained resources. As a possible trade off, even minimal measures such as the use of a simple score, brief standardised comments or tick boxes for different aspects of a research proposal may already give applicants some basic guidance about the perceived quality of their research. Other more differentiated, tiered feedback approaches may involve the submission of an expression of interest or a preliminary proposal to obtain initial feedback before a full submission—an approach that helps balance efficiency with fairness and distributes the workload for applicants and reviewers while preserving formative value.

Criticism lies at the heart of scientific progress; without it, there is no learning. If PPIE is to enrich research, it must be meaningful and reciprocal. At a time when public trust in science is more vulnerable than ever, such efforts are not optional—they are essential.

## Data Availability

No datasets were generated or analysed during the current study.
